# Molecular Characteristics and Distribution of Adult Human Corneal Immune Cell Types

**DOI:** 10.3389/fimmu.2022.798346

**Published:** 2022-02-23

**Authors:** Yanxiu Li, Joyce Jeong, Weitao Song

**Affiliations:** ^1^ National Clinical Research Center for Geriatric Diseases, Xiangya Hospital of Central South University, Changsha, China; ^2^ Eye Center of Xiangya Hospital, Central South University, Changsha, China; ^3^ Hunan Key Laboratory of Ophthalmology, Changsha, Hunan, China; ^4^ College of Literature, Science, and the Arts, University of Michigan, Ann Arbor, MI, United States

**Keywords:** single-cell transcriptome, corneal immune cells, MregDC, antiviral CD8^+^ T cells, chemotactic

## Abstract

**Background:**

The limbus is located at a 2-mm-wide area between the bulbar conjunctiva and the cornea and has been suggested to be the niche of corneal epithelial stem cells and immune cells. Like the skin and intestines, the cornea is also an important mucosal surface, and immune cells on the cornea play critical roles in immune surveillance to ensure barrier surface homeostasis and protection from various environmental damage and infections. Single-cell RNA sequencing (scRNA-seq) analysis of protein tyrosine phosphatase receptor type C positive (PTPRC^+^) hematopoietic cells from the corneal limbus could provide a single cell atlas of all the immune cell subsets.

**Methods:**

We performed single-cell RNA sequencing to generate transcriptomic profile for 804 sort-purified hematopoietic cells from the corneal limbus of three healthy donors.

**Results:**

Our analysis identified a primary transcriptomic pattern for multiple immune cell subtypes, including naive T cells, antiviral effector CD8^+^ T cells, and innate immune cells such as IDO1^+^ mature regulatory dendritic cells (mregDCs), macrophages, monocytes, and basophils in the human corneal limbus.

**Conclusion:**

Overall, single-cell transcriptomic analysis of limbal immune cells suggested the possible contribution of these cells on the adaptive and innate immune response of the human cornea.

## Introduction

Ocular surface diseases represent a huge medical need and are a substantial burden to many families (1). Due to the limited treatments for these diseases, investigation of the immune system of the ocular surface is crucial. The ocular surface, which comprises the cornea, limbus conjunctiva, and tear film, plays a key role in the visual system. Among these structures, the cornea is an avascular and transparent anterior surface that, together with the lens focus, allows light to be transmitted to the retina for visual processing (2). Like other mucosal surfaces, the cornea is the surface between the inner tissue and the external environment. It is responsible for protecting the eyes against microbes through innate and adaptive immune systems. The cornea has five distinctive layers, and the corneal limbus is considered an important niche of epithelial cells and immune cells on the ocular surface (3). Generally, the physical barrier formed by corneal epithelial cells can prevent pathogens from invading, while the flow of tears along with blinking washes pathogens away. Most importantly, the immune cells on the human cornea provide crucial mucosal immune response to prevent infections and damage.

Human and animal studies have observed altered immune cell distributions or functions in particular eye diseases, such as dry eye and eye allergies (4–7). The ocular surface, like other mucosal tissues, can recruit a variety of immune cells to render protection and homeostatic regulation (8, 9). However, dysfunction of the immune cells on the ocular surface would cause disruption of the corneal epithelial barrier function and ocular surface homeostasis (7, 10, 11). Therefore, ocular surface immune profiling studies are important to understand ocular surface homeostasis and related diseases. So far, the immune cell types on the cornea of mice have been covered and investigated well. T cells, dendritic cells (DCs), macrophages, mast cells, natural killer cells, γδ T cells, and innate lymphoid cells (ILCs) have been investigated on murine cornea (12). However, the present knowledge surrounding corneal immune cells has been primarily limited to murine data, while the composition of immune cells on the human cornea requires more investigation. Due to the rich distribution of capillaries and lymphatic vessels that serve as the entry and exit portals for various immune cells, the corneal limbus is home to immune cells that reside in both the central and peripheral corneal regions (13). In the present study, to better investigate the immune cell types on the human cornea, we performed single-cell RNA sequencing(scRNA-seq) to generate transcriptomic profile for sort-purifiedhematopoietic cells from the corneal limbus of three healthy donors. Unbiased analyses identified seven immune cell types, including innate and adaptive immune cell types. This transcriptomic map of healthy human corneal immune cells can be utilized to better understand the immune response and regulation of the human cornea and help lead toward potential cellular and immunotherapy approaches. Furthermore, transcriptomic information can provide functional insight into the mechanisms of diseases such as viral infections, wound repair, and autoimmune diseases like allergies.

## Materials and Methods

### Human Samples and Single-Cell Isolation

The collection of human corneal tissue was approved by the Ethics Committee of Xiangya Hospital. Fresh corneal tissue was peeled gently from three healthy adults using surgical forceps under a stereoscope. To isolate the corneal limbus, the central cornea was carefully removed. After isolating the corneal limbus tissues, we dissociated the tissues and obtained single-cell suspensions based on a previous protocol (14). The corneas were briefly chopped in the media and then digested using collagenase A, dispase II, and DNAse I at 37°C for 20 min. The cell suspension was then sorted for live PTPRC^+^ cells using a FACSAria III cell sorter (BD Biosciences, Franklin Lakes, NJ, USA) at 4°C into 1.5-ml DNA low-binding Eppendorf tubes containing medium. Sorted purified samples were collected and pelleted for processing with 10X Genomics v2.

### Genomics scRNA-seq and Data Analysis

Single cells suspended in phosphate-buffered saline (PBS) were loaded into a single-cell instrument from the 10X Genomics system. A barcoded cDNA library was constructed using Single-Cell 3′ mRNA Kit (v2, 10X Genomics). On the Illumina NovaSeq 6000 platform, all libraries (paired-end) were sequenced after passing quality tests. The 10X Genomics single-cell transcriptome sequencing data were integrated from the pooled cells of three donors and processed with the Cell Ranger Single Cell software suite version 1.3 (https://support.10xgenomics.com) as described previously (15). The output data were analyzed using the SeqGeq genomic tool version 9.0 (FlowJo, LLC, Ashland, OR, USA). Principal component analysis (PCA) reduction (15 dimensions) was performed, followed by an unbiased *t*-distributed stochastic neighbor embedding (*t*-SNE) dimensionality reduction. We then performed clustering with a *k*-means filtering of *k* = 57 to cluster the cells into seven populations based on the variability in the PCA. The PhenoGraph algorithm (16) was used to identify the distinct ILC progenitor clusters. The seven clusters identified by PhenoGraph were overlaid onto the *t*-SNE map. The Cluster Explorer plug-in was used to characterize the immunophenotype of each cluster. Mean cluster transcript expression plots and expression heatmaps of differentially expressed genes were acquired by conducting the Color Mapping program. Gene Ontology (GO) (molecular function) and Kyoto Encyclopedia of Genes and Genomes (KEGG) pathway enrichment analyses were performed using Metascape (http://metascape.org) (17) and Reactome (https://reactome.org).

### Flow Cytometry Analysis and Reagents

For the validation of individual markers, the cells were analyzed on a NovoCyte flow cytometer (Agilent Technologies, Santa Clara, CA, USA). Hematopoietic cells were isolated as PTPRC^+^ (anti-human CD45 and QA17A19) cells using a FACSAria III cell sorter (BD Biosciences). All the antibodies used in this study were from BioLegend (San Diego, CA, USA), unless specified otherwise: anti-human CD3 (UCHT1), anti-human CD8 (SK1), anti-human CD4 (A161A1), anti-human IFN-γ (MD-1), anti-human granzyme B (GB11), anti-human granzyme A (CB9), anti-human CD96 (NK92.39), anti-human CD74 (LN2), anti-human CCR7 (G043H7), anti-human IDO1 (V50-1886; BD Biosciences), anti-human HLA-A, HLA-B, and HLA-C (W6/32), anti-human HLA-DR (L243), anti-human HLA-DQ (HLADQ1), anti-human CD164 (67D2), anti-human IL-10 (JES3-19F1), anti-human CXCL16 (22-19-12), anti-human TGF-β1 (TW4-9E7; BD Biosciences), anti-human perforin 1 (DG9; antibodies-online Inc., Pottstown, PA, USA), anti-human IL-32 (373821; R&D Systems, Minneapolis, MN, USA), and anti-human CXCL2 (rabbit IgG polyclonal; biorbyt, Cambridge, UK). Anti-human CXCR6 monoclonal antibody and recombinant human CXCL16 were purchased from R&D System. For intracellular cytokine staining, freshly isolated cells were stained with surface markers and then reactivated for 4 h with 10 ng/ml phorbol myristate acetate (PMA) and 1 µM ionomycin (Sigma, St. Louis, MO, USA) prior to fixation and permeabilization with cytokine staining. The data were then analyzed using FlowJo version 10 (TreeStar) and GraphPad Prism.

### Chemotaxis Assay

Human corneal T-cell migration was evaluated using a 24-well Transwell plate (5.0-μm pore size; Corning, Corning, NY, USA) as described previously (18). Freshly sorted purified corneal CD3^+^ T cells were washed once with RPMI 1640 medium and then placed in 100 μl T-cell medium in the top chamber of the Transwell plate with or without the addition of 1 μg/ml of anti-human CXCR6 antibody. The bottom chamber of the Transwell plate contained chemokine *CXCL16* (100 ng/ml) or the supernatant from the CD164^+^ corneal innate immune cell culture medium (600 μl). After 90 min incubation at 37°C in a 5% CO_2_ atmosphere, the top chamber was removed and the number of T cells that had migrated into the bottom chamber was counted using flow cytometry. Migration rate was determined by calculating the percentage of input cells that migrated into the lower chamber.

### Statistical Analysis

For statistical analysis of normally distributed continuous variables between two groups, an unpaired Student’s *t*-test was used. Significance between multiple groups was determined using one-way ANOVA. A *p*-value <0.05 was considered to be significant in this study. All data were presented as the mean ± standard error of the mean (SEM).

### Available Data

Processed data from scRNA-seq are available at the ArrayExpress database in the European Nucleotide Archive EMBL-EBI, with accession ID E-MTAB-11027.

## Results

### Identification of Immune Cell Populations in the Corneal Limbus

**Figure 1:**
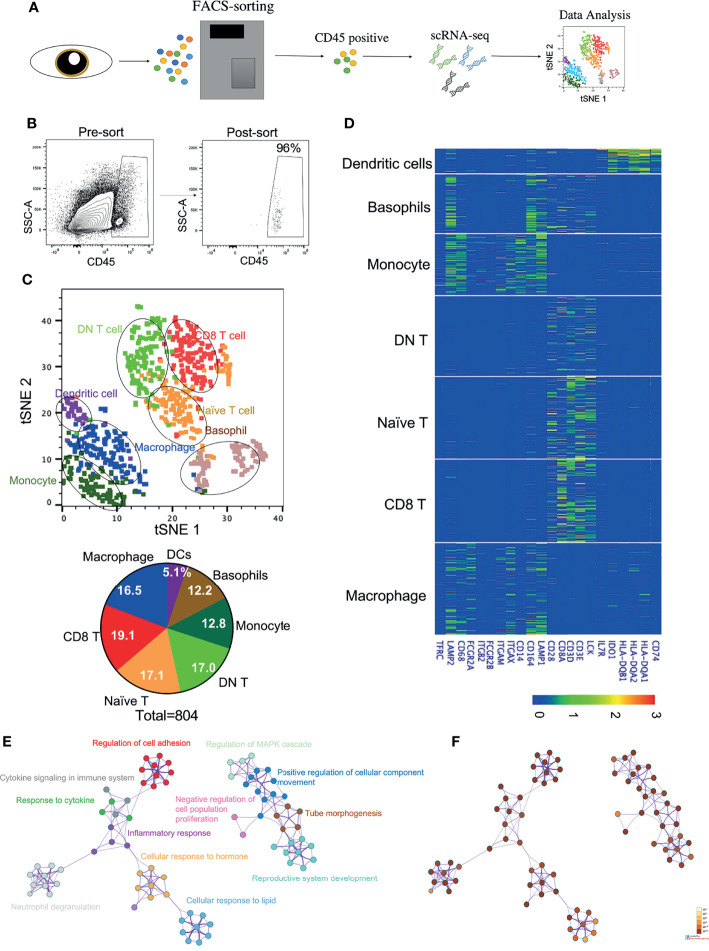
Identification of immune cell types in the cornea limbus. **(A)** Schematic of the single-cell RNA sequencing (scRNA-seq) workflow. The corneal limbus was gently digested to a single-cell suspension, enriched for hematopoietic cells by sorting PTPRC^+^ cells, and then scRNA-seq was performed. The data were then analyzed. **(B)** Gating strategy for sorting. Live CD45^+^ cells were selected. **(C)** Unbiased *t*-distributed stochastic neighbor embedding (*t*-SNE) clustering was used to determine the cell types and the frequency of the different immune cell types. **(D)** Heatmap of the specific marker genes for each cell type in scRNA-seq. **(E)** Network of enriched terms colored by cluster identity, where nodes that share the same cluster identity are typically close to each other. **(F)** Network of enriched terms colored by *p*-value, where terms containing more genes tend to have a more significant *p*-value.

To determine the transcriptome profiles of all immune cell subsets in the cornea, we decided to perform transcriptomic analysis on the sorted purified hematopoietic cells from the human corneal limbus. As our focus is primarily on immune cells, we used PTPRC, a marker for hematopoietic cells, to distinguish immune cells from other cell types such as epithelial and stromal cells in the cornea. We performed fluorescence-activated cell sorting (FACS) to isolate PTPRC^+^ hematopoietic cells from the corneal limbus after removal of the corneal endothelium and central cornea. Human adult corneas were excised from three healthy male donors (18, 50, and 78 years old). The collected cells were dissociated and subjected to the 10X Genomics platform for scRNA-seq (**Figures 1A, B** **)**. Transcriptome profiling of 804 cells was performed after passing quality control. These cells were embedded, and seven major cell clusters were revealed using unsupervised *t*-SNE and unbiased clustering (**Figure 1C**). Clusters 1–7 were determined to comprise macrophages, naive T cells, double-negative (DN) T cells, CD8^+^ T cells, monocytes, basophils, and DCs, respectively, based on specific marker genes (**Figure 1D**). For instance, macrophages and monocytes were identified by *CD68*, while DCs were identified by the high expressions of the *HLA* subtypes and *CD74*. The percentage of each immune cell cluster was determined (**Figure 1C**). The data suggested that innate immune cells occupied around half of the clusters, while the other half was identified as T lymphocytes. Specifically, CD8^+^ T cells (19.1%) and macrophages (16.5%) were the predominant subsets in adaptive and innate immune cells, respectively. To gain more insight into the function of corneal immune cells, a total of 1,217 most differentially expressed genes were used to generate enriched ontology clusters with Metascape (**Figures 1E, F** **)**. Two major clustering trees were visualized, and biological pathways such as response to cytokine, hormone, and lipid were associated with inflammatory response. Another putative biological function is cellular movement and development regulation. Overall, the genes expressed on the corneal immune cells were enriched in inflammatory response and cellular development. In addition, pathway analysis using Reactome also supported these differentially expressed genes being enriched in multiple immune response pathways such as cytokine regulation, MHC class II antigen presentation, and neutrophil degranulation (**Supplementary Figures S1A, B** **)**.

### Antiviral Effector CD8^+^ T Cells Are the Predominant T-Lymphocyte Subset

**Figure 2:**
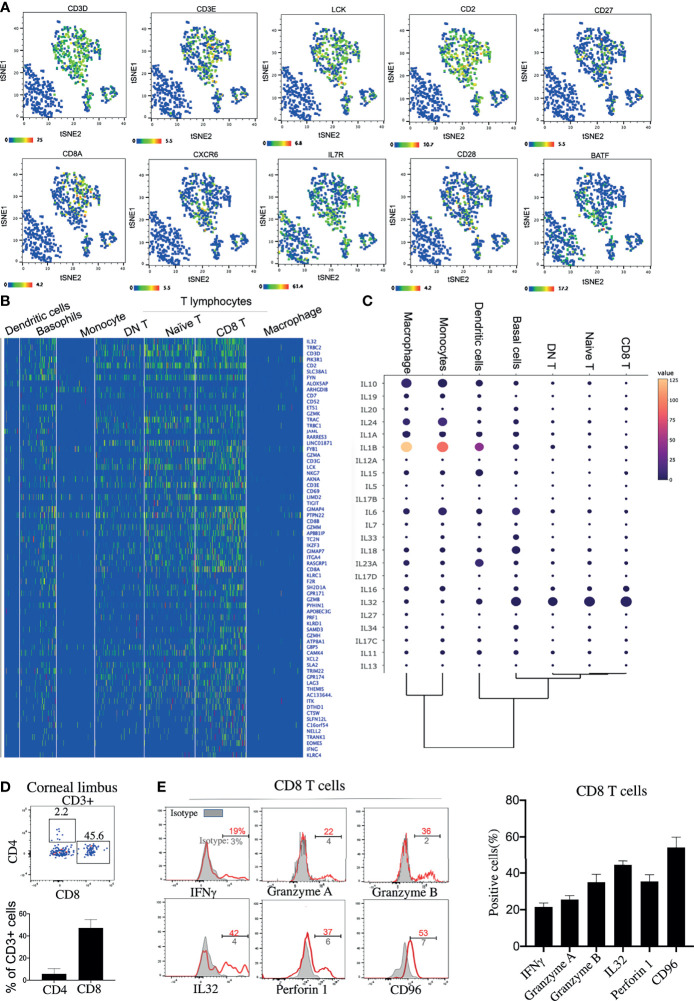
Antiviral effector CD8^+^ T cells are a predominant lymphocyte subset on the cornea. **(A)** Feature *t*-distributed stochastic neighbor embedding (*t*-SNE) plot showing the expressions of marker genes enriched on corneal T cells. **(B)** Heatmap of the top expressed genes in the corneal T-cell subsets. **(C)** Dot plot showing the expressions of genes of the different cytokines (*rows*) on each cluster (*columns*). The *color of each dot* represents the average log-scaled expression of each gene across all cells of a given cluster. The *size of the dot* represents the fraction of cells in the cluster in which transcripts for that gene were detected. **(D)** Expressions of CD8 and CD4 proteins on corneal CD3^+^ T cells by flow cytometry **(E)** Expressions of selected cytokines and surface proteins on corneal CD8^+^ T cells by flow cytometry.

Three clusters of T lymphocytes were identified by the high expressions of specific T-lymphocyte markers: *CD3E*, *CD3D*, and *LCK* (**Figure 2A**). However, B lymphocytes were barely detected on the corneal limbus (**Supplementary Figure S1C**). Among the clusters, three T-lymphocyte subsets—naive T cells, DN T cells, and CD8^+^ T cells—were identified based on specific markers (**Figures 2A–C** **)**. In line with a previous study (19), *CD4* mRNA was hardly detected on the corneal limbus, according to our scRNA-seq data. However, it has been reported that the mRNA expression of CD4 did not match the protein expression (20), and detectable protein levels of CD4 were confirmed by flow cytometry (**Figure 2D**). Interestingly, the antiviral capacity of the CD8 T cell subset was identified based on the high expressions of the activation markers *CD69* and *Lag3* and the antiviral genes *GZMA*, *GZMB*, *PRF1*, *IFNG*, and *IL32* (**Figures 2B, C** **)**. Additionally, corneal limbal CD8^+^ T cells expressed the surface receptor *CD96*, which has been considered as a co-stimulatory receptor that enhances CD8^+^ T-cell activation in humans (21). We subsequently confirmed the expressions of IFN-γ, granzyme A, granzyme B, IL-32, perforin 1, and CD96 from the corneal limbal CD8^+^ T cells based on protein level by flow cytometry (**Figure 2E**). Therefore, we proposed that CD8^+^ T cells specifically act as functional effectors in controlling viral spread on the cornea. Another important T-lymphocyte subset is naive T cells, which expressed *CD28*, *CD27*, and *IL7R* (**Figures 2A, B** **)**. Furthermore, naive T cells also specifically expressed an important pioneer chromatin modifier, *BATF*, which is an essential transcriptional factor in regulating the differentiation of effector CD8^+^ T cells (22). Therefore, we suggested that corneal limbal BATF^+^ naive T cells could differentiate into antiviral effector CD8^+^ T cells. Unexpectedly, we also found a DN T-cell subset located in the corneal limbus that contained a few γδ T cells (**Supplementary Figure S1C**), which requires further investigation in the future. In summary, we proposed that corneal limbal CD8 T cells are the major subset of immune cells preventing the corneal tissue from becoming virally infected by producing cytokines and cytotoxic granules, including IFN-γ, granzyme A, granzyme B, IL-32, and perforin 1. Furthermore, effector CD8^+^ T cells are likely to be differentiated from BATF-expressing naive T cells.

### Anti-Inflammatory Macrophages and Monocytes Recruit Naive T Cells by Secreting *CXCL16*

**Figure 3:**
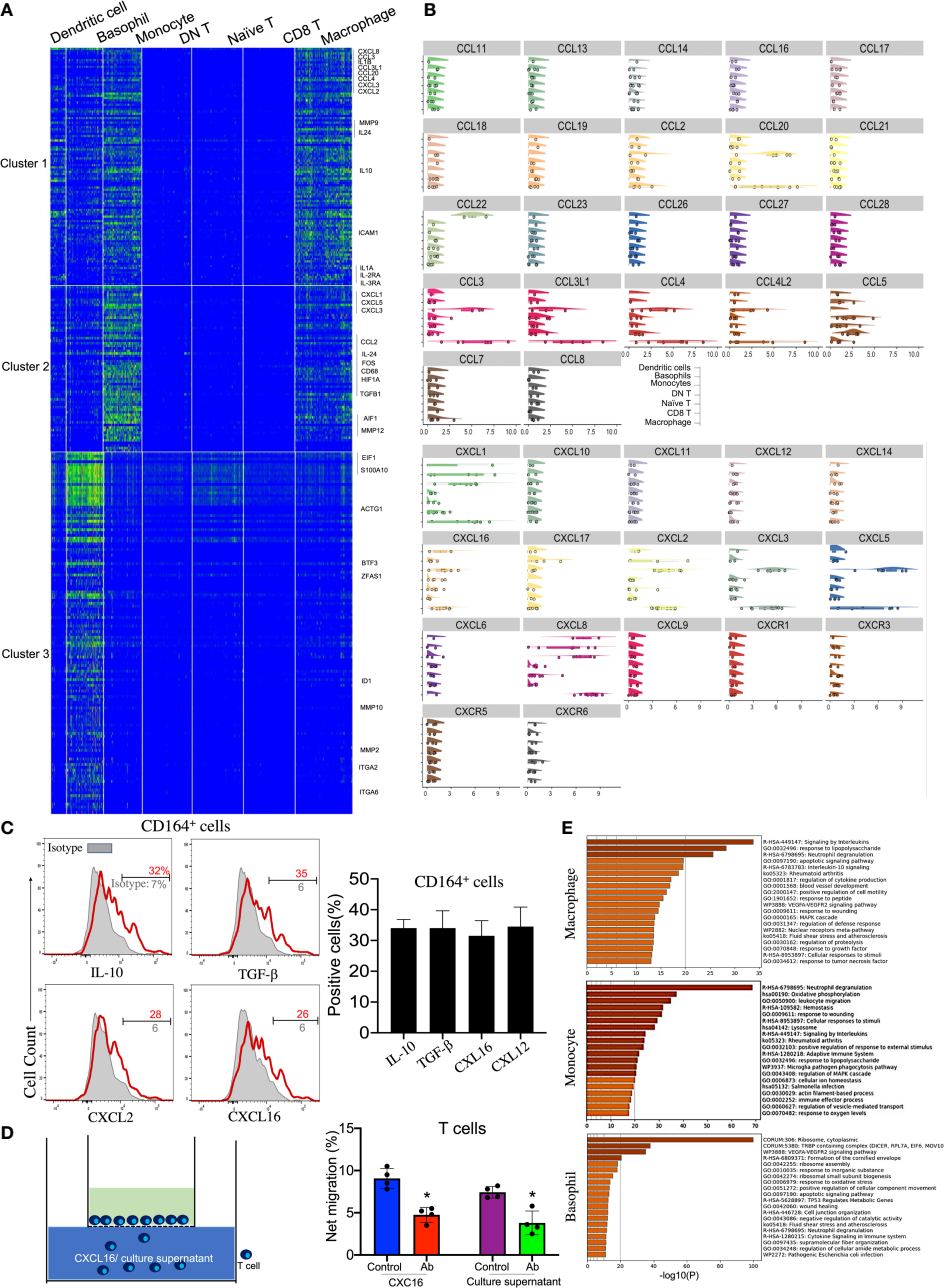
Immunoregulatory macrophages and monocytes recruit naive T cells by secreting CXCL16. **(A)** Heatmap of the top expressed genes in corneal macrophages, basophils, and monocytes. **(B)** Violin plot of the expressions of chemokine (C–X–C motif) ligands and chemokine (C–X–C motif) receptors in each cluster. **(C)** Expressions of IL-10, TGF-β, CXCL16, and CXCL12 on CD164^+^ corneal innate immune cells by flow cytometry. **(D)** Chemotactic activity of corneal CD3+ T cells to 100 ng/ml CXCL16 or the cell culture supernatant was determined with the Transwell migration system. **(E)** Pathway enrichment analysis of the differentially expressed genes in corneal macrophages, basophils, and monocytes. *Significant differences (P < 0.05, Student’s t test) from control groups.)

Accordingly, four distinct clusters of innate immune cells—monocytes, macrophages, basophils, and DCs—were observed and annotated through specific markers (**Figures 1D**, **3A**, and **Table 1**). Among these cell subsets, basophils were identified by the high expressions of *Lamp1/2* and *CD164*. Macrophages expressed specific genes such as *CD14*, *CD68*, and *FCGR2A/B*. Interestingly, various chemokines from the C–X–C motif ligand family, including *CXCL1*, *CXLC2*, *CXCL3*, *CXCL5*, *CXCL8*, and *CXCL16*, were expressed on these innate immune cell types, especially monocytes and macrophages (**Figures 3A, B** **)**. Among the CXCL chemokine family, CXCL16 is the ligand for CXCR6 which is also highly expressed on corneal naive T cells. It has been reported that *CXCL16* could induce the chemoattraction of CXCR6^+^ human skin T cells (23), so we were eager to investigate whether corneal innate immune cells could recruit naive T cells by generating *CXCL16*. Additionally, all three innate immune cells highly expressed *CD164* mRNA, which is generally expressed by granulocytes, based on the scRNA-seq data (**Figure 1D**). We also confirmed the production of *CXCL16* from corneal limbal CD164^+^ cells by flow cytometry (**Figure 3C**). To conduct an *in vitro* chemotaxis assay, we placed the recombinant CXCL16 or supernatant from the CD164^+^ corneal limbal immune cell culture medium in the lower chambers and the sorted purified corneal CD3^+^ T cells in the presence of isotype or anti-CXCR6 antibody in the upper chambers. The chemotaxis data (**Figure 3D**) suggested that both recombinant *CXCL16* and the cell culture supernatant can attract T cells from the upper chamber. In addition, blocking CXCR6 using antibodies could significantly inhibit the chemoattraction of T cells toward the recombinant *CXCL16* or the cell culture supernatant in the lower chamber. Therefore, the data indicated that corneal limbal innate immune cells can potentially recruit naive T cells into the corneal limbus and generate antiviral effector CD8^+^ T cells downstream. Furthermore, the generation of anti-inflammatory cytokines such as IL-10 and TGF-β from monocytes and macrophages indicated that they play an immunoregulatory role in the immune response of the human corneal limbus (**Figures 2C** and **3A, B**). Wound healing of the cornea is a multistep process with four overlapping but distinct stages: hemostasis, inflammation, proliferation, and remodeling. Corneal limbal macrophages and monocytes produced the chemokines *CXCL1*, *CXCL2*, and *CXCL8*, which are important for the inflammation and proliferation stages of wound healing (**Figures 3B, C** **)**. Furthermore, both macrophages and monocytes could respond to wounding based on the KEGG pathway and GO annotation analyses (**Figure 3E**). It also suggested that corneal macrophages, monocytes, and basophils are capable of the degranulation and regulation of cytokine production. Thus, besides attracting naive T cells, cornea limbal innate immune cells also have the potential to suppress the immune response and aid in wound healing.

**Table 1:** Gene list of each cluster from **Figure 3A**.

Cluster 1	Cluster 2	Cluster 3
*CCL3L1*	*CCL2*	*APOBEC3A*
*CCL20*	*CXCL5*	*KRT17*
*CCL3*	*CD14*	*S100A2*
*CCL4*	*TGFBI*	*KRT6A*
*CCL4L2*	*CTSD*	*PERP*
*IL1B*	*THBS1*	*TACSTD2*
*EREG*	*FCER1G*	*KRT14*
*IL10*	*SPP1*	*KRT5*
*CTSL*	*S100A9*	*CLDN4*
*CXCL8*	*RNASE1*	*SFN*
*CXCL3*	*MT1G*	*SPRR2A*
*CXCL2*	*PPBP*	*EMP1*
*INHBA*	*S100A8*	*MMP3*
*MMP9*	*TYROBP*	*ADIRF*
*CREG1*	*CTSB*	*MAL2*
*G0S2*	*SERPINB2*	*KRT16*
*SOD2*	*IL24*	*CLDN1*
*APOE*	*CHI3L1*	*DSP*
*TIMP1*	*LGALS1*	*ACTG1*
*SRGN*	*FTH1*	*CTSV*
*PTGS2*	*CXCL3*	*SPRR1B*
*C15orf48*	*FTL*	*MMP12*
*ACSL1*	*SLC11A1*	*SLPI*
*IER3*	*NAMPT*	*S100A14*
*ABCA1*	*MS4A7*	*ANXA1*
*HSP90AA1*	*CD300E*	*CD24*
*FNIP2*	*LAPTM5*	*LYPD3*
*OGFRL1*	*HIF1A*	*KRT19*
*SOCS3*	*CD68*	*IGFBP3*
*PDE4DIP*	*CAPG*	*GPRC5A*
*PLAUR*	*DMXL2*	*CSTB*
*FTL*	*LCP1*	*LMO7*
*CXCL1*	*AIF1*	*APOD*
*ATP2B1*	*MT1H*	*DSG3*
*NAMPT*	*AKR1B1*	*FGFBP1*
*TNFRSF1B*	*GRN*	*PTGDS*
*CD93*	*NPC2*	*ALDH1A3*
*BCL2A1*	*CXCL1*	*S100A10*
*CYP1B1*	*PLD3*	*TM4SF1*
*ANXA5*	*PLAUR*	*HSPA5*
*BASP1*	*BRI3*	*DSC2*
*ICAM1*	*C5AR1*	*SPRR2D*
*NFKBIA*	*S100A4*	*AKR1C2*
*IL24*	*LYZ*	*MT2A*
*PSAP*	*FOS*	*ELF3*
*ATP13A3*	*MT1F*	*S100A16*
*ETS2*	*TMEM176B*	*CLU*
*NRP2*	*PLIN2*	*HSPB1*
*BCAT1*	*FCGRT*	*S100A6*
*DSE*	*SPI1*	*S100A11*
*PDE4B*	*TUBB*	*EZR*
*CD83*	*VCAN*	*ANXA2*
*FABP5*	*PKM*	*RND3*
*SLC16A10*	*GAPDH*	*SERPINE2*
*PPP1R15A*	*LYN*	*TPT1*
*NINJ1*	*ASAH1*	*DCN*
*CTSZ*	*ATP6V1F*	*CAMK2N1*
*CD63*	*PILRA*	*ID1*
*SAMSN1*	*GPNMB*	*LGALS3*
*STC1*	*TNFRSF1B*	*CRYAB*
*CTSB*	*RNF130*	*CD55*
*SMOX*	*AQP9*	*NDRG1*
*PLEK*	*CEBPB*	*RPL24*
*CD44*	*CTSZ*	*C11orf96*
*IL1A*	*KYNU*	*MMP1*
*ZEB2*	*OAZ1*	*AKR1C1*
*OSBPL8*	*PID1*	*DSG1*
*DUSP4*	*MAFB*	*KLF5*
*RCAN1*	*DUSP1*	*PHLDA2*
*WTAP*	*SERF2*	*CLDN7*
*FCER1G*	*CYBA*	*JUP*
*GJB2*	* *	*GSTP1*
*SLC7A11*	* *	*SULT2B1*
*VIM*	* *	*LDHA*
*GADD45B*	* *	*SERPINB5*
*PLA2G7*	* *	*PPP1R14B*
*MPC2*	* *	*KRT13*
*SNAPC1*	* *	*RPS8*
*IL1RN*	* *	*TNFRSF11B*
*FLNA*	* *	*C4orf3*
*ITGB8*	* *	*HMOX1*
*CTSS*	* *	*CD9*
*FCGR2A*	* *	*ITGA2*
*CD68*	* *	*YBX3*
*IL2RA*	* *	*TSC22D1*
*MPZL1*	* *	*SELENOM*
*DAB2*	* *	*C6orf132*
*KYNU*	* *	*RPS13*
*MIR3945HG*	* *	*SLC2A1*
*SMS*	* *	*DENND2C*
*PFKFB3*	* *	*C19orf33*
*DNAJB6*	* *	*HMGA1*
*TNIP3*	* *	*KRT12*
*TNFAIP3*	* *	*CD59*
*MMP14*	* *	*KRT6B*
*ZFYVE16*	* *	*CAST*
*IL3RA*	* *	*SH3BGRL3*
*SNX10*	* *	*DSTN*
*MALAT1*	* *	*PLS3*
*EPB41L3*	* *	*RPL5*
*CTNNB1*	* *	*RPS6*
*NRP1*	* *	*HSP90B1*
*CTSH*	* *	*IGFBP6*
*GSTO1*	* *	*NMB*
*PILRA*	* *	*RPS24*
*MAP4K4*	* *	*PLAU*
*PDPN*	* *	*HSP90AB1*
*SERPINA1*	* *	*GIPC1*
*KLF6*	* *	*AQP3*
*GRAMD1A*	* *	*NACA*
*ASAH1*	* *	*RPL37A*
*EGR1*	* *	*PRDX5*
*MS4A7*	* *	*ISG20*
*TCF4*	* *	*SDCBP2*
*SPP1*	* *	*CAV1*
*MCL1*	* *	*RPS5*
*DOCK4*	* *	*RPL7*
*ARL4C*	* *	*COL17A1*
*TRAF1*	* *	*RPL26*
*PTPN1*	* *	*RPL7A*
*SEMA6B*	* *	*RPS18*
*ATP6V1F*	* *	*DST*
*OTUD1*	* *	*RPLP1*
*GK*	* *	*RPL8*
*TLR2*	* *	*HEBP2*
*PTPRE*	* *	*GGCT*
*ABL2*	* *	*BTF3*
*PPIF*	* *	*RPL18*
*MAP2K3*	* *	*RPL36*
*NR4A2*	* *	*RAP2B*
*GYPC*	* *	*RPL15*
*SQSTM1*	* *	*EIF1*
*EHD1*	* *	*PPP1CB*
*ASPH*	* *	*ANXA11*
*CFLAR*	* *	*TNFRSF12A*
*THBD*	* *	*RPS12*
*SAT1*	* *	*CEBPD*
*SLC5A3*	* *	*PTPN13*
*JUN*	* *	*ZFAS1*
*MIR155HG*	* *	*RHOD*
*AKR1B1*	* *	*RPS3A*
*LHFPL2*	* *	*ITGA6*
*CSF2RA*	* *	*RPL21*
*NANS*	* *	*RPS28*
*MARCKS*	* *	*FXYD3*
*RILPL2*	* *	*RPS7*
*PID1*	* *	*RPS23*
*ATP6V0B*	* *	*SDC1*
*SERPINB9*	* *	*DRAP1*
*DRAM1*	* *	*S100A13*
*CSF2*	* *	*CSTA*
*TNFAIP8*	* *	*HOPX*
*FTH1*	* *	*SCEL*
*GPR183*	* *	*MALL*
*CCL18*	* *	*RPLP0*
*CD109*	* *	*MMP10*
*TALDO1*	* *	*RPL11*
*TNFRSF4*	* *	*RPL35*
*UBE2D1*	* *	*MMP2*
*MAFB*	* *	*KRT15*
*LITAF*	* *	*RACK1*
*CEBPB*	* *	*RPL41*
*TNIP1*	* *	*RPL37*
*MMP19*	* *	*VDAC2*
*SDCBP*	* *	*RPS27A*
*IQGAP1*	* *	*RPL35A*
*GPAT3*	* *	*LAMB3*
*ATP6V1B2*	* *	*RPL10A*
*MGLL*	* *	*RPL14*
*GLA*	* *	*RPL4*
*ATP8B4*	* *	*RPL19*
*MT1X*	* *	*PPDPF*
*THBS1*	* *	*MAL*
*PDIA3*	* *	*GUK1*
*RGCC*	* *	*RPL13*
*EIF5*	* *	*RPL30*
*CRIM1*	* *	*FOSL1*
*IVNS1ABP*	* *	*DSC3*
*GLUL*	* *	*ZFAND5*
*LPXN*	* *	*RPL22*
* *	* *	*RPL3*
* *	* *	*RPS15*
* *	* *	*RPL12*
* *	* *	*RPL32*
* *	* *	*RPL39*
* *	* *	*IL6*
* *	* *	*RPL18A*
* *	* *	*NPM1*
* *	* *	*RPL23A*
* *	* *	*CAV2*
* *	* *	*RPL29*
* *	* *	*RPL27*
* *	* *	*RPL9*
* *	* *	*SELENOK*
* *	* *	*NNMT*
* *	* *	*SLK*
* *	* *	*RPL27A*
* *	* *	*RPL23*
* *	* *	*MUC16*
* *	* *	*RPS10*
* *	* *	*RPL34*
* *	* *	*FAM129B*
* *	* *	*RAN*
* *	* *	*RPLP2*
* *	* *	*YWHAQ*
* *	* *	*CNN3*
* *	* *	*RPS4X*
* *	* *	*H3F3B*
* *	* *	*SOD3*
* *	* *	*RPS21*
* *	* *	*SERP1*
* *	* *	*MUC22*
* *	* *	*LGALSL*
* *	* *	*PRDX1*
* *	* *	*MYO6*
* *	* *	*RPL6*
* *	* *	*KRT18*
* *	* *	*PFDN2*
* *	* *	*KRT10*
* *	* *	*CHCHD2*
* *	* *	*RPL13A*
* *	* *	*SPINT2*
* *	* *	*SEC61G*
* *	* *	*RPS2*
* *	* *	*RPS14*
* *	* *	*ABI3BP*
* *	* *	*RPS15A*
* *	* *	*RPS25*
* *	* *	*CCDC85B*
* *	* *	*USP53*
* *	* *	*AFDN*
* *	* *	*CALM2*
* *	* *	*ARF4*
* *	* *	*LUM*
* *	* *	*EEF1A1*
* *	* *	*KLF4*
* *	* *	*PITX1*
* *	* *	*CLTA*
* *	* *	*RAC1*
* *	* *	*ACTN4*
* *	* *	*RPS3*
* *	* *	*EEF1B2*
* *	* *	*CDH1*
* *	* *	*MT-ND4L*
* *	* *	*COL6A2*
* *	* *	*CYCS*
* *	* *	*NEAT1*
* *	* *	*RIOK3*
* *	* *	*RPS11*
* *	* *	*RPSA*
* *	* *	*CAPN2*
* *	* *	*LAMC2*
* *	* *	*RPS19*
* *	* *	*CST3*
* *	* *	*RPL38*
* *	* *	*IGFBP4*
* *	* *	*EPS8L1*
* *	* *	*EEF2*
* *	* *	*TNFAIP6*
* *	* *	*NQO1*
* *	* *	*SLC25A3*
* *	* *	*TMSB10*
* *	* *	*PKP1*
* *	* *	*RPL28*
* *	* *	*FAM83A*
* *	* *	*LMAN1*
* *	* *	*POLR1D*
* *	* *	*MYL12B*
* *	* *	*COX7A1*
* *	* *	*CLMP*
* *	* *	*ECM1*
* *	* *	*GOLGA4*
* *	* *	*RAB11A*
* *	* *	*SDF2L1*
* *	* *	*IGFBP2*
* *	* *	*PPL*
* *	* *	*RPS9*
* *	* *	*RPS26*
* *	* *	*TGM1*
* *	* *	*RPS29*
* *	* *	*YWHAE*
* *	* *	*PRNP*
* *	* *	*CCND1*
* *	* *	*SLC39A14*
* *	* *	*RPL36AL*
* *	* *	*RAB11FIP1*
* *	* *	*CNBP*
* *	* *	*TPM4*
* *	* *	*RTN4*
* *	* *	*SOX4*
* *	* *	*PDLIM4*
* *	* *	*ADGRF1*
* *	* *	*ACTB*
* *	* *	*NUPR1*
* *	* *	*UBC*
* *	* *	*SET*
* *	* *	*YWHAZ*
* *	* *	*BZW1*
* *	* *	*HES1*
* *	* *	*HNRNPA1*
* *	* *	*HERPUD1*
* *	* *	*COX7A2*
* *	* *	*MT-CYB*
* *	* *	*ST14*
* *	* *	*RPL36A*
* *	* *	*NCL*
* *	* *	*FAU*
* *	* *	*PHLDA3*
* *	* *	*SF3B6*
* *	* *	*PAX6*
* *	* *	*ERO1A*
* *	* *	*ARF6*
* *	* *	*FST*
* *	* *	*TUBB4B*
* *	* *	*EIF2S2*
* *	* *	*NAP1L1*
* *	* *	*PPIA*
* *	* *	*RPL10*
* *	* *	*CEACAM6*
* *	* *	*SDC4*

### Corneal Limbal Dendritic Cells Are IDO1^+^ mregDCs

**Figure 4:**
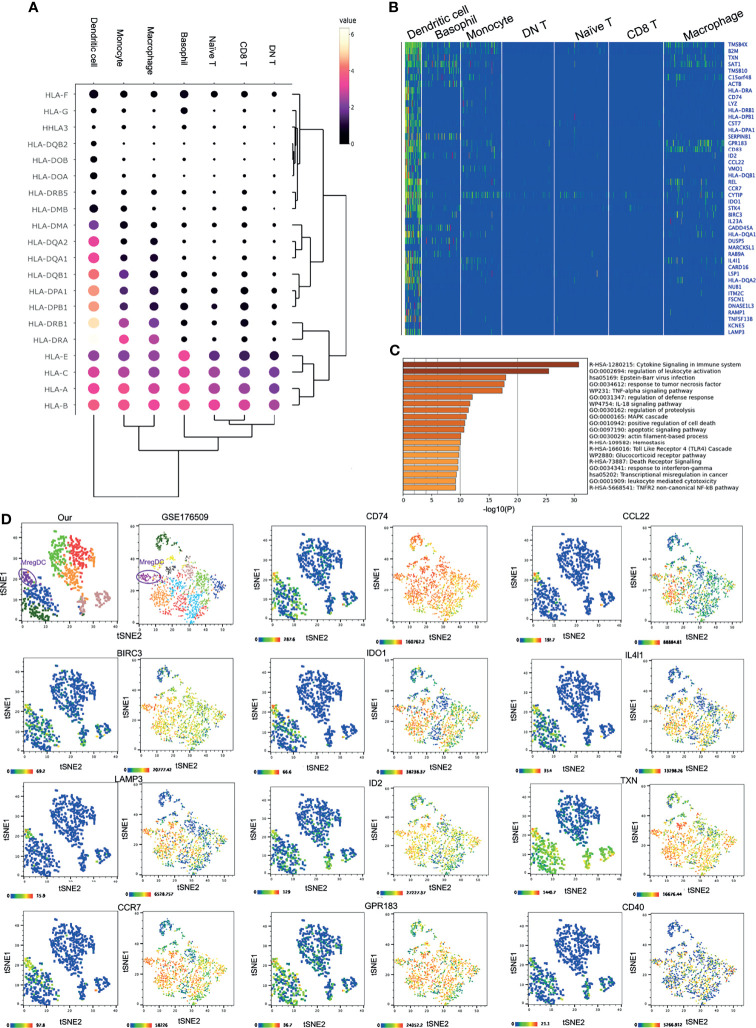
IDO1^+^ mature regulatory dendritic cells (mregDCs) are the major antigen-presenting cells on the cornea. **(A)** Dot plot showing the expressions of genes of the HLA subtypes (*rows*) in each cluster (*columns*). The *color of each dot* represents the average log-scaled expression of each gene across all cells of a given cluster. The *size of the dot* represents the fraction of cells in the cluster in which transcripts for that gene were detected. **(B)** Heatmap of the top expressed genes in corneal dendritic cells. **(C)** Pathway enrichment analysis of the differentially expressed genes in corneal dendritic cells. **(D)** The *t*-distributed stochastic neighbor embedding (*t*-SNE) plot showing comparative expressions of mregDC-specific signature genes from our data and those of Nakamizo et al. (20).

DCs comprise the unique cell type among the corneal innate immune cells. Although other immune cells such as macrophages and monocytes could express the major HLA subtypes, such as *HLA-A/B/C/E*, *HLA-DRA*, and *HLA-DRB1*, corneal limbal DCs expressed various other subtypes, including *HLA-DPB1*, *DPA1*, *DQB1*, *DQA1/2*, and *DMA* (**Figures 4A, B**). Therefore, it is suggested that DCs function as the major antigen-presenting cells connecting the innate and adaptive immune systems on the human cornea. The KEGG pathway and GO annotation analyses were performed by using Metascape and showed that corneal DCs participate in cytokine signaling in the immune system and regulate leukocyte activation (**Figure 4C**). Surprisingly, we found that the corneal limbal DCs are mregDCs, which displayed high expressions of the markers *LAMP3* and *BIRC3 *(24) (**Figure 4B** and **Supplementary Figure S2A**). This new subset of DCs has recently been identified in human skin mucosal tissues. To better compare the mregDCs between the corneal limbus and the skin, we reanalyzed other publicly available skin datasets (GEO accession no. GSE176509) (24) and identified skin mregDCs using the same methods of *t*-SNE analysis and clustering. We found that, except for *LAMP3* and *BIRC3*, both corneal and skin mregDCs highly expressed *CCR7*, *CD74*, *ID2*, *GPR183*, *CCL22*, *IL4l1*, *CD40*, and *TXN* (**Figure 4D** and **Supplementary Figure S2**). In addition, corneal limbal mregDCs also expressed high levels of the pro-inflammatory cytokines IL1B, IL-15, and IL-23A, which are associated with mucosal inflammation (24). MregDCs have also been found to be involved in cytokine signaling and regulation of leukocyte activation (**Figures 2C** and **4C, D**). The analysis indicated that mregDCs potentially play a pro-inflammatory role in the immune response of the cornea. Interestingly, consistent with recent papers (25), we also found that the mregDCs in both the skin and corneal limbus specifically expressed indoleamine-2,3-dioxygenase (IDO1), a counter-regulatory and tolerogenic molecule (**Figure 4D**). Although there were several subsets of DCs identified in the skin, only one corneal limbal DC subset was detected on the cornea limbus, and this DC subset belongs to mregDCs. Furthermore, corneal limbal mregDCs do not only act as antigen-presenting cells but also participate in regulating immune tolerance by coordinating with other innate immune cells.

## Discussion

On the ocular surface, the cornea is a unique and highly specialized tissue that is avascular and transparent in order to allow light to be transmitted for vision. Recent studies have identified cell types in the cornea using scRNA-seq; however, they primarily paid attention to epithelial and stromal cells, the major cell types in the cornea (19, 25, 26). In this study, we concentrated on the immune cell types and observed seven distinct immune cell clusters that differed in function. Overall, we reported a primary scRNA-seq analysis of the human corneal innate and adaptive immune cell types, including antiviral effector CD8^+^ T cells, naive T cells, and innate immune cells such as IDO1^+^ mregDCs, macrophages, monocytes, and basophils, providing a detailed map of corneal immune cell function. It should be noted that, because aging could lead to changes in the immune system, such as the distribution and number of immune cells (27, 28), despite using similar numbers of cells from each donor, the wide donor-to-donor age variation could affect the results to a certain extent. We identified subtype-specific transcriptional factors and surface markers for the different immune cell types. Through flow cytometry, but not scRNA-seq, a few CD4^+^ T cells were detected on the human cornea in a steady state. In addition, human corneal naive T cells highly expressed *BATF*, which has been proven to be crucial for the differentiation of effector CD8^+^ T cells in a steady state (22) and during viral infection (29). Therefore, BATF is likely to be an important regulator for the differentiation of naive T cells into antiviral effector CD8^+^ T cells in the human cornea. The co-stimulatory receptor *CD96* and the activation markers *Lag3* and *CD69* were highly expressed on CD8^+^ T cells (21, 30, 31). Furthermore, several antiviral cytokines and cytotoxic granules, such as IFN-γ, granzyme A, granzyme B, perforin 1, and IL-32, were produced by corneal CD8^+^ T cells, which further confirmed that CD8^+^ T cells in the human cornea have the ability to fight against viruses even at a steady state. Therefore, we believe that CD8^+^ T cells are a significant immune cell type that prevents virus infection in the human cornea, which is consistent with a previous finding on mouse corneas (32). Furthermore, it has been proven that severe acute respiratory syndrome coronavirus 2 (SARS-CoV-2) was detected on the human cornea (33, 34). As is known, CD8^+^ T cells are crucial to the prevention of SARS-CoV-2 infection (35), so it can be considered that corneal CD8^+^ T cells may also contribute to protecting the eyes from SARS-CoV-2 infection. We also proved that, from *in vitro* studies, CXCR6-expressing naive T cells are attracted by the specific chemokine *CXCL16* produced by corneal innate immune cell types, including macrophages, DCs, and monocytes. This result indicated that *CXCL16* is not only required for the colon’s immune system (36) but is also important in maintaining the immune response of the cornea. Because we did not detect regulatory T cells in the cornea, we were eager to search for other immunoregulatory immune cell types. Interestingly, sequencing and flow cytometry data indicated that macrophages and monocytes are dominant cell types that produce IL-10 and TGF-β, which are known anti-inflammatory cytokines that suppress the immune response. Meanwhile, they also express various chemokine genes such as *CCL3*, *CCL20*, *CCL4*, *CC3L1*, *CCL4L2*, *CXCL2*, *CXCL8*, and *CXCL5*. We believe that these innate immune cells can recruit and regulate distinct cell types by generating chemokines. Analyses of KEGG pathway and GO annotation also indicated that macrophages, monocytes, and basophils could degranulate and be involved in wound healing. Another interesting innate immune cell type comprise DCs, the major antigen-presenting cells in the cornea. DCs displayed their ability to present antigens by highly expressing various HLA subtype genes such as *HLA-DQA*, *HLA-DPA*, and *HLA-DPB*. Recently, researchers have identified a small subset of skin DCs called mregDCs with high expressions of *BIRC3*, *LAMP3*, *IL15*, *CD40*, and *CCR7*, and this subset has been thought to be associated with wound healing and the exacerbation of atopic dermatitis (24, 37, 38). It is surprising to find that corneal DCs also belong to mregDCs, with high expressions of the unique markers *BIRC3* and *LAMP3*. Furthermore, we compared the corneal and skin mregDCs by reanalyzing the data from public scRNA-seq and unexpectedly found that there are high levels of similarity between the mregDCs from these two mucosal tissues. The three major pro-inflammatory cytokine genes expressed on corneal DCs were *IL1B*, *IL-15*, and *IL-23A.* Consistently, all three cytokines were produced by skin DCs and considered to be associated with atopic dermatitis and psoriasis. Additionally, GO enrichment analysis of the upregulated genes in DCs suggested that corneal DCs participate in cytokine signaling and regulation of leukocyte activation. Consequently, we suggested that the cytokines IL-1β, IL-15, and IL-23a produced by corneal mregDCs could be crucial to ocular inflammation. Intriguingly, both corneal and skin mregDCs also expressed IDO1, which is a heme-containing enzyme that can suppress T-cell response. Additionally, IDO1-expressing DCs may provide an immunoregulatory network by promoting the development of regulatory T cells (39). Therefore, DCs could play a crucial role in maintaining and regulating a dynamic balance between pro- and anti-inflammatory signals.

In summary, the goal of this study was to provide crucial information regarding all the immune cell types located in the adult human cornea limbus. Identification of the different corneal immune cell types using transcriptomic analysis can help in understanding the eye immune network. In addition, this study revealed the genes/pathways of immune cells that could lead to improvements in immunotherapies for corneal disease and wound repair of the corneal limbus.

## Data Availability Statement

The processed data of single-cell RNA-seq are available at the ArrayExpress database in European Nucleotide Archive EMBL-EBI with accession ID E-MTAB-11027.

## Ethics Statement

The studies involving human participants were reviewed and approved by the Ethics Committee of Xiangya Hospital. The patients/participants provided written informed consent to participate in this study.

## Author Contributions

WS conceptualized, acquired funding, and supervised this study. YL performed sample collection, single-cell dissociation, and library preparation. Data were processed, curated, and visualized by YL. YL, JJ and WS drafted the manuscript. All authors contributed to the article and approved the submitted version.

## Funding

This study was supported by the National Nature Science Fund of China (81974132), National Key R&D Program of China (2021YFA1101202), Hunan Provincial Health Commission (20220702839) and National Nature Science Fund of China (81770927).

## Conflict of Interest

The authors declare that the research was conducted in the absence of any commercial or financial relationships that could be construed as a potential conflict of interest.

## Publisher’s Note

All claims expressed in this article are solely those of the authors and do not necessarily represent those of their affiliated organizations, or those of the publisher, the editors and the reviewers. Any product that may be evaluated in this article, or claim that may be made by its manufacturer, is not guaranteed or endorsed by the publisher.

## Supplementary Material

Supplementary Figure S1 Supporting data for cluster analysis. **(A)** Overview of relevant pathways involved in the immune system obtained from Reactome. **(B)** Most relevant pathways sorted by p-value for differentially expressed genes. **(C)** Feature t-SNE plot showing expression of other important marker genes.

Supplementary Figure S2 The expression of DCs signature genes and chemokine receptors. **(A)** Violin plot of expression of DCs signature genes in each cluster. **(B)** Violin plot of expression of CC chemokine receptor genes in each cluster.
